# A Rare Case of a Primary Non-parasitic Splenic Cyst Removed via Laparoscopic Intervention

**DOI:** 10.7759/cureus.75263

**Published:** 2024-12-07

**Authors:** Mubashir Rafique, Muhammad Moiz Muzaffar, Fatima Rauf, Muhammad Haris Khan, Muhammad Usama Tahir, Muhammad Hanif, Huma Sabir Khan

**Affiliations:** 1 Cardiology, Pinderfields Hospital, Wakefield, GBR; 2 Surgical Unit II, Benazir Bhutto Hospital, Rawalpindi Medical University, Rawalpindi, PAK; 3 Paediatrics, Worcestershire Acute National Health Service (NHS) Hospital Trust, Worcester, GBR; 4 Ear, Nose and Throat (ENT), Sunderland Royal Hospital, Sunderland, GBR; 5 General Surgery, Rawalpindi Medical University, Rawalpindi, PAK

**Keywords:** cystic swelling, laproscopic surgery, left hypochondrial swelling, non-hydatid splenic cyst, non-parasitic splenic cyst, primary splenic cyst, spleenomegaly, splenic cystectomy, splenic cysts

## Abstract

Splenic cysts are differentiated into primary and secondary cysts based on epithelial lining. Primary non-parasitic epithelial splenic cysts are extremely rare. We report a case of a 24-year-old male with left hypochondrial swelling with no history of abdominal trauma. His hydatid serology was negative. A CT scan showed massive splenomegaly due to a large, relatively thin-walled cystic lesion with possible thin internal septation near the lower pole of the spleen. The entire cyst and its intact wall were excised laparoscopically. The cyst's nature and eventual laparoscopic removal, with its parenchyma preserved, make this a unique case.

## Introduction

Splenic cysts with epithelial linings are called primary splenic cysts, which can be parasitic or non-parasitic [1]. The parasitic cysts most commonly include hydatid cysts caused by Echinococcus granulosus. Non-parasitic cysts are rare and include epidermal, neoplastic, and vascular cysts [1,2]. Cysts that do not have well-defined epithelial lining are called secondary cysts and arise from trauma or infection. In this case, a 24-year-old male was diagnosed with a primary splenic cyst and underwent laparoscopic cystectomy.

## Case presentation

A 24-year-old man came to our outpatient department complaining of swelling in the left hypochondrium, which he first noticed four years ago. It was non-painful, but he was having slight discomfort intermittently. The swelling was initially about the size of a tennis ball but gradually increased. There was an associated dragging sensation and undocumented weight loss. There was no history of abdominal trauma. His past medical and surgical history was notable only for a right-sided hernioplasty performed in 2014. The patient was vitally stable, and abdominal examination revealed a firm, non-tender mass arising from the left hypochondrium reaching up to the umbilicus. It was approximately 12x14 cm in size with a dull percussion note. The possibility of a hydatid cyst was ruled out by negative hydatid serology.

**Figure 1:**
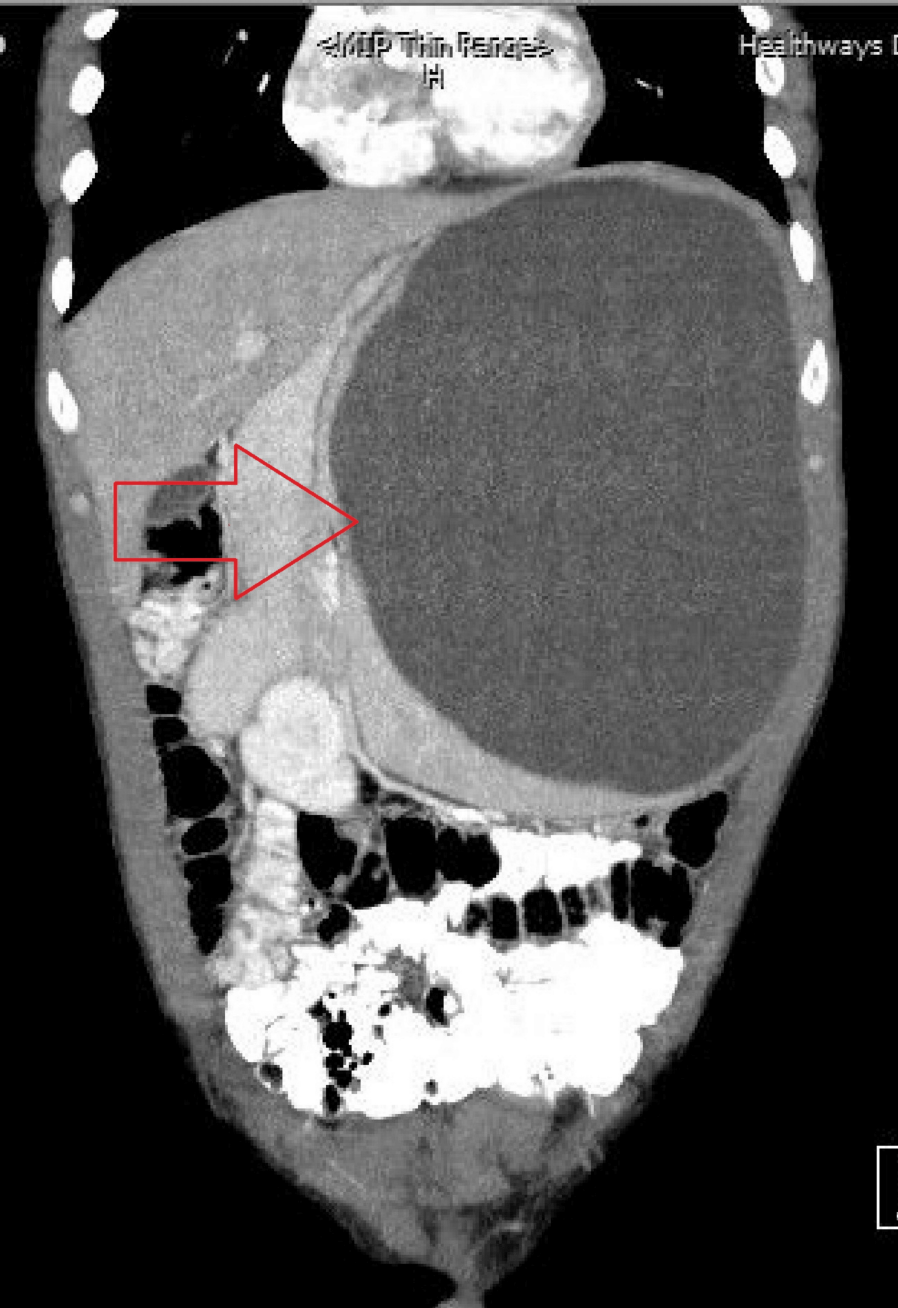
CT scan of the patient (anteroposterior view) Arrow showing splenic cyst on CT scan (anteroposterior (AP) view).

Baseline investigation results are shown in Table 1, which revealed a normal white cell count (WCC) as well as normal liver and renal function. Viral serology was negative. Determination of anti-echinococcus immunoglobulin (Ig)G antibodies by enzyme-linked immunosorbent assay (ELISA) was done, and they were found to be negative. CT scan showed massive splenomegaly due to a large, relatively thin-walled cystic lesion with possible thin internal septation near the lower pole of the spleen. The lesion measured 24 × 20 × 16 cm (craniocaudal x transverse x anteroposterioror CC, AP, TX), and the estimated volume of the cyst fluid was 3840 ml. It was causing a mass effect with displacement of the surrounding bowel and left kidney to the right. Tiny specs of calcifications were seen in its posterosuperior wall, but there was no internal fat. The lesion showed no abnormal contrast enhancement (see Figure 1).

**Table 1:** Initial investigation results WCC: White cell count; RCC: Red cell count; HCT: Hematocrit; MCV: Mean corpuscular volume; MCH: Mean corpuscular hemoglobin; MCHC: Mean corpuscular hemoglobin concentration; AST: Aspartate aminotransferase; SGOT: Serum glutamic-oxaloacetic transaminase; ALT: Alanine aminotransferase; SGPT: serum glutamate pyruvate transaminase.

Investigation	Value	Units	Range
Hemoglobin	11.7	g/dL	12.0-16.0
WCC	10.6	10^3^/uL	4.0-11.0
Platelets	179	10^3^/Ul	150.0-450.0
RCC	4.53	10^6^/uL	3.50-5.50
HCT	37.8	%	35.0-50.0
MCV	83.4	Fl	76.0-96.0
MCH	25.8	pg	26.0-32.0
MCHC	31.0	g/dL	32.0-36.0
Neutrophils	10.0	10^3^/uL	2.5-7.0
Lymphocytes	0.5	10^3^/uL	1.5-4.5
AST SGOT	33.8	u/L	Upto 45
Bilirubin total	1.6	mg/dl	Upto 1.2
Alkaline phosphatase	120.5	U/L	Adults (32-92)
ALT SGPT	20.8	u/L	Upto 43
Sodium	142	mEq/L	135-145
Potassium	4.31	mEq/L	3.5-5.0
Chloride	106	mEq/L	95-108
Serum urea	20.42	mg/dl	15-50
Serum creatinine	1.2	mg/dl	0.7-1.2

**Figure 2:**
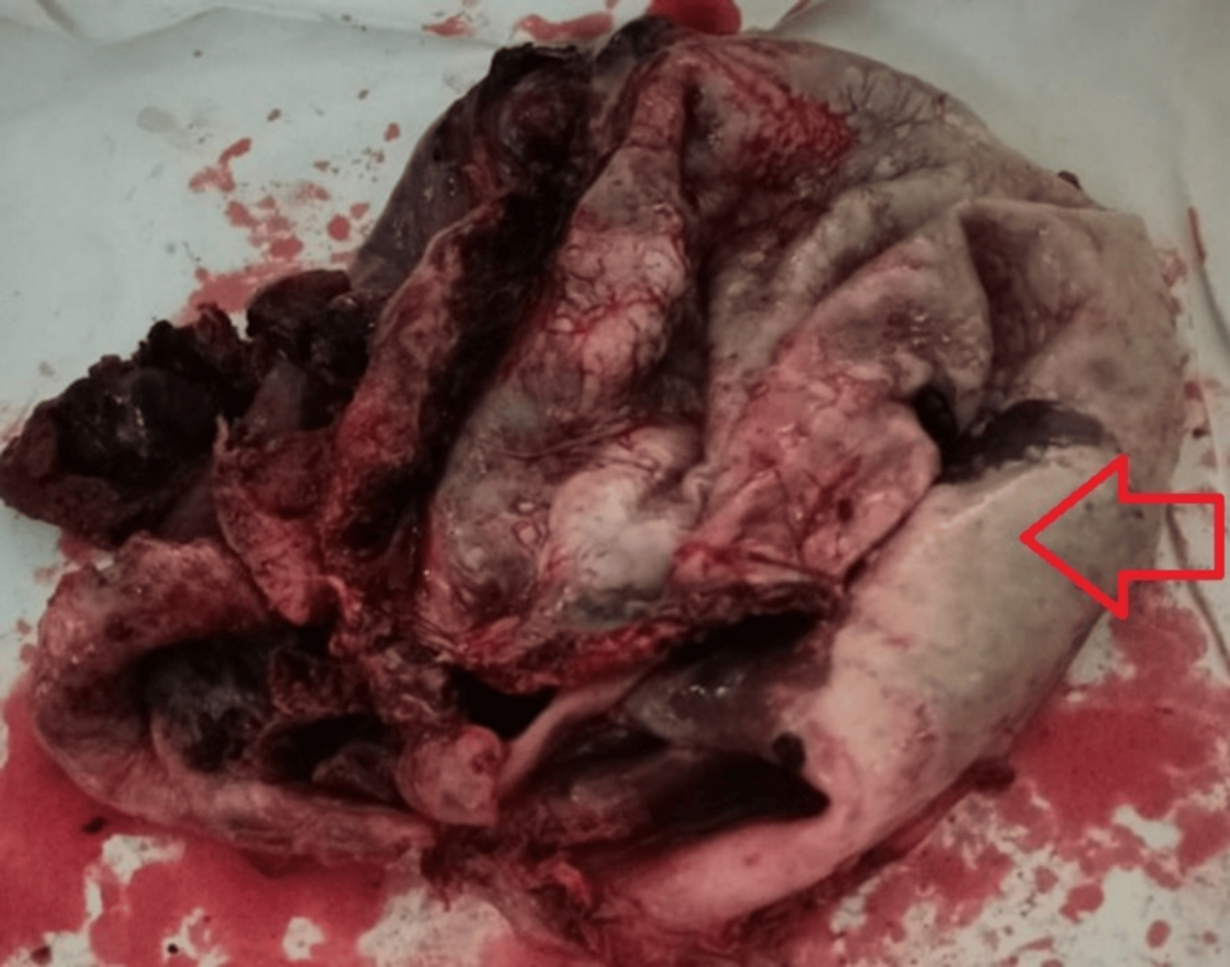
Arrow showing excised splenic cyst after fluid removal

The patient underwent laparoscopic cystectomy. Per-operative findings included a large cyst at the lower pole of the spleen extending up to the diaphragm and displacing the pancreas, stomach, and left kidney. The cyst contained approximately 5L of turbid fluid and was excised completely from the splenic tissue with an intact wall (see Figure 2).

The splenic fluid analysis showed increased total leukocyte count (TLC), red cell count, and neutrophils. The histopathology report showed a cyst wall with a denuded lining. Few areas were lined by single-cell layers of flat to cuboidal mesothelial and epithelial cells. The fibrovascular stroma was infiltrated by lymphocytes, eosinophils, and plasma cells. Fibrosis and foreign body type giant cell reactions were also seen. The immunohistochemistry report was positive for cytokeratin AE1AE, calretinin, and human bone marrow endothelium marker-1 (HBME-1). A final diagnosis of a true, non-parasitic splenic cyst was made. The patient was discharged on the fifth postoperative day without any complications.

## Discussion

Splenic cysts are rare, with a reported incidence of only 0.07-0.3 % [3,4]. They are classified into primary or secondary cysts based on the presence or absence of an epithelial lining. Primary cysts, called true cysts, are divided into parasitic and non-parasitic cysts. The non-parasitic cysts comprise epidermal, vascular, and neoplastic cysts. The secondary cysts (pseudocysts) usually arise secondary to trauma, infarction, and infection [1,2]. A splenic cyst can have varying symptoms depending on its size. It can be asymptomatic or cause symptoms like early satiety, dragging sensation, or pain in the left flank radiating to the left shoulder. Large cysts can also compress the surrounding organs [5]. Hydatid serology is used to rule out parasitic cysts. Ultrasound scan (USS), CT scan, and MRI are used as diagnostic modalities for a splenic cyst and can determine whether the cyst is unilocular or multilocular, its wall thickness, or the presence of any calcification [6]. Treatment depends upon the size of the cyst. If the cyst is <5 cm and asymptomatic, it is managed conservatively. The surgical approach is preferred if the cyst is >5 cm and is symptomatic [5]. Surgical options include percutaneous drainage, marsupialization, fenestration, laparoscopic or open cystectomy, or splenectomy (complete or partial) [5,7]. The laparoscopic technique is more suited for peripherally located cysts and is associated with less morbidity in terms of wound complications, faster recovery, and preserved splenic function [8]. Vaccination and counselling are necessary to prevent overwhelming post-splenectomy infection (OPSI) if the entire spleen is removed [9].

## Conclusions

Primary non-parasitic splenic cysts are very rare. Fluid analysis and other modern tests can aid in diagnosing the specific subtype of splenic cyst. The advent of new laparoscopic techniques in conjunction with radiological findings mean that every effort can be made to conserve the splenic architecture in order to prevent immediate or late post-op complications.

## References

[1] Morgenstern L (2002). Nonparasitic splenic cysts: pathogenesis, classification, and treatment. J Am Coll Surgeons.

[2] Fowler RH (1913). Cysts of the spleen: a pathological and surgical study. Ann Surg.

[3] Hammouda SB, Mabrouk S, Bellalah A, Maatouk M, Zakhama A, Njim L (2022). Large splenic epithelial cyst: a rare presentation. Int J Surg Case Rep.

[4] Senn AS, Bauer RC, Heigl A, Rosenberg R (2022). 23-year old man with a long history of abdominal pain, nausea and vomiting: case report of a splenic cyst. Int J Surg Case Rep.

[5] Hansen MB, Moller AC (2004). Splenic cysts. Surg Laparosc Endosc Percutan Tech.

[6] Robertson F, Leander P, Ekberg O (2001). Radiology of the spleen. Eur Radiol.

[7] Smith ST, Scott DJ, Burdick JS, Rege RV, Jones DB (2001). Laparoscopic marsupialization and hemisplenectomy for splenic cysts. J Laparoendosc Adv Surg Tech A.

[8] Tagaya N, Oda N, Furihata M, Nemoto T, Suzuki N, Kubota K (2002). Experience with laparoscopic management of solitary symptomatic splenic cysts. Surg Laparosc Endosc Percutan Tech.

[9] Tahir F, Ahmed J, Malik F (2020). Post-splenectomy sepsis: a review of the literature. Cureus.

